# The Impact of Physical Distancing in the Pandemic Situation: Considering the Role of Loneliness and Social Brain

**DOI:** 10.3389/fpsyg.2022.861329

**Published:** 2022-03-21

**Authors:** Rosalba Morese, Claudio Longobardi

**Affiliations:** ^1^Faculty of Communication, Culture and Society, Università della Svizzera italiana, Lugano, Switzerland; ^2^Faculty of Biomedical Sciences, Università della Svizzera italiana, Lugano, Switzerland; ^3^Department of Psychology, University of Turin, Turin, Italy

**Keywords:** physical distancing, loneliness, physical contact, social inclusion, isolation, social neuroscience

## Abstract

The new normal has made social distancing a new way of experiencing sociality. Social neuroscience has for a long time been concerned with studying the beneficial effects of social relationships, of physical contact. It is known that physical contact activates neurophysiological processes that reduce the perception of discomfort and even physical pain. So in the absence of physical contact, our social brain may be modulated differently when we are with others. But what could be the long-term effects of this normality? This mini-review focuses on highlighting with the support of social neuroscience evidence such as isolation, distancing can affect people’s health.

## Introduction

The World Health Organization [WHO] (2020) has defined physical distancing as a useful behavior to limit the spread of COVID-19. This means keeping a distance of at least 1 m and also avoiding spending a lot of time in places with many people; thus, physical distancing can prevent transmission of the pandemic and protect those most at risk, even after the vaccination campaign is over (Jarvis et al., 2020). Thorp (2020) raised considerations and questions about public health and epidemiology issues about the long-term effects on mental health and social wellbeing. What might be the impact of physical distance in this context?

Kumar and Salinas (2021) summarized in a review how social isolation, loneliness, and the long-term effects of social distancing may impact in population’s health, cognitive impairment, social processes, and also neurodegenerative diseases (Morese et al., 2018, 2020; Palermo et al., 2018, 2020; Morese and Palermo, 2020).

## Loneliness

Loneliness is defined as the subjective perception of feeling socially isolated and is often described as dissatisfaction with the discrepancy between desired and actual social relationships (Perlman and Peplau, 1984; Holt-Lunstad et al., 2015). While loneliness and social isolation may arise from the experience of social isolation, they are not synonymous and appear to be independent constructs. Loneliness, in fact, refers to a subjective perception that can be determined by an introspective assessment of the subject, whereas social isolation concerns a lack or deprivation in relation to a person’s objectively observable social network. Loneliness tends to be associated with physical and mental disorders (Groarke et al., 2020; McClelland et al., 2020; Winterton et al., 2020). Rico-Uribe et al. (2018) in a recent meta-analysis, also showed that loneliness is a risk factor for all-cause mortality because it is strongly associated with depression. This was found in both men and women, although slightly more often in men. From an evolutionary theory perspective (Cacioppo et al., 2014), social isolation may pose a risk to individual survival. According to theorists, the feeling of loneliness has evolved as an internal alarm signal that prompts individuals to reconnect and restore social ties. The quality of relationships and the narrowing of the social network may be triggers that activate the feeling of loneliness, while physical contact may be the element of the external environment that deactivates the internal alarm signal. Although the way physical contact occurs between people is influenced by cultural aspects (Heatley Tejada et al., 2020), in line with evolutionary theory, it can be observed how in all cultures physical contact is used by people to establish interpersonal closeness and stay connected to their social network. Social involvement and close relationships tend to promote psychological adjustment in individuals by positively influencing mood and affect regulation, thus promoting better self-perception (Fabris et al., 2020; Lin et al., 2020; Marengo et al., 2021). Along these lines, physical contact, understood as positive contact, attests to the subject’s involvement in a social network and satisfies bonding and close relationship needs, reducing feelings of loneliness. Experimental evidence shows that administering physical contact reduces perceptions of loneliness, especially for single participants (Heatley Tejada et al., 2020). Physical contact may make the subject feel more accepted by others, which contributes to experiencing a sense of safety in the relationship with the other and developing a perception of oneself as a valuable and loved subject, which promotes prosocial and protective behaviors in the relationship with others (Jakubiak and Feeney, 2017). In fact, several lines of evidence suggest that physical touch is associated with greater relationship satisfaction (Gleason et al., 2008; Jakubiak and Feeney, 2017), cooperative, and prosocial behaviors (Guéguen and Fischer-Lokou, 2003; Kraus et al., 2010; Jakubiak and Feeney, 2017). By making the subject feel socially included and accepted by others, physical contact could positively influence the subject’s self-esteem and mood, increasing focus on interpersonal relationships and building close relationships (Jakubiak and Feeney, 2017; Heatley Tejada et al., 2020). Overall, then, these observations seem to suggest that physical contact, especially affectionate contact, promotes individuals’ psychological wellbeing. It likely makes subjects feel included in close relationships and social networks, thus reducing feelings of loneliness.

Unfortunately, the feeling of loneliness has greatly increased since the beginning of 2020, changing people’s daily lives. It is known that the cause of this change is the spread of a new coronavirus (SARS-CoV-2). There are now numerous studies that have examined the impact of foreclosure on mental health (Galić et al., 2020), as well as the relationship to certain constructs such as depression and poorer sleep quality, increased stress, social isolation, loneliness, and anxiety for their health and economic aspects (Rania and Coppola, 2021).

Notably, the most notorious effects were initially found in older adults (Armitage and Nellums, 2020) and research has highlighted that social isolation in older adults increases the risk for psychological and medical problems (Cipolletta and Gris, 2021). Loneliness increases the occurrence of post-traumatic symptoms and, more importantly, both received and perceived social support may be protective factors against psychological problems. Based on these findings, strategies have been proposed to assist older people by maintaining social support and a sense of belonging and reducing loneliness, such as technological support (Parisi et al., 2021).

## Loneliness and Social Brain

Lam et al. (2021) associated loneliness with increased morbidity. Humans have developed evolutionarily rooted neural, hormonal, and genetic mechanisms to live with others; they have a need to belong to society, to feel included. Belonging to social groups is a need of all individuals. It also has an evolutionary advantage, because thanks to the support of others, the chances of survival increase. In line with this, some authors have studied how the brain activates the reward network during behaviors that promote group cooperation and affiliation (Fehr and Gächter, 2000; Tomasello, 2009; Morese et al., 2016; Rabellino et al., 2016; Gerfo et al., 2019). Courtney and Meyer (2020) studied 43 individuals using functional magnetic resonance imaging (fMRI) and asked them to perform a reflection task to elicit activation associated with thinking about close people and acquaintances under the following experimental conditions: Self, nominated close persons, nominated acquaintances, and known personalities. In addition, participants indicated their subjective closeness to each person and their sense of loneliness in a questionnaire. The authors examined neural responses in a specific brain area normally associated with self-representation, the medial prefrontal cortex (mPFC). This brain area is associated with cognitive processes related to identity, including in cases of psychopathology (for example, borderline personality disorder, see Bozzatello et al., 2019). Results showed that participants who had less social contact (i.e., were more lonely) had altered self-other mapping in social brain regions. The importance of being socially connected to others is well known, but the brain mechanisms involved in relationships with others, particularly in the case of loneliness, have not been sufficiently studied or clarified. This research suggests how the social brain appears to map our interpersonal ties, and changes in the social map may influence other cognitive and social processes at different stages of life.

## Physical Contact

On the other side of loneliness, a physical contact is an important form of social support known to play a protective role in reducing neural, physiological, and neuroendocrine responses to pain, stress, and inflammation (Montoya et al., 2004; Ozbay et al., 2007; Thomas and Kim, 2021). Social support as physical contact effectively reduces physiological responses to social stress, even painful responses to social exclusion and experiences in which negative emotions are felt and some brain areas are recruited that are similar to those of physical pain (Morese et al., 2019a). The effect of physical contact on the emotional brain is possible thanks to the modulation of the activity of brain areas concerned with emotions and emotion regulation (Longobardi et al., 2020; Morese and Longobardi, 2020). Interpersonal relationships provide an important context for emotion regulation and promote better adjustment across the lifespan (Morese et al., 2019b; Lindsey, 2020; Morese and Longobardi, 2020). Along these lines, physical contact tends to reduce stress in adults and children, and physical contact that is perceived as affectionate, thus signaling caring and love, leads individuals to feel more secure and in a more intimate relationship with others, which promotes feelings of emotional connection and inclusion in relationships with others (Jakubiak and Feeney, 2017; Suvilehto et al., 2019). It is possible, then, that physical contact is associated with deeper and more intimate emotional relationships in which individuals feel accepted and included, contributing to individuals’ positive adjustment and reduced psychological distress. This seems to be particularly relevant for those at risk of social exclusion, such that their network of social contacts shrinks and they experience feelings of loneliness.

Recently, in line with the new evidence on the effects and consequences of social exclusion and isolation, authors (Gryksa and Neumann, 2022) presented in a review paper the possible short- and long-term consequences of social restrictions imposed to avoid contagion and their impact on mental health, with particular emphasis on the immune system. The immune system is strongly modulated by the stress response (all types of stress, including social stress) and is dysregulated in mental disorders (e.g., depression, anxiety); lack of social support may exacerbate this immune system dysregulation, but oxytocin may reduce the stress response. In this context, the authors suggest that oxytocin may be a potential treatment option for reducing psychological and physical stress levels and mental health (Gryksa and Neumann, 2022).

## Conclusion

The lack of social support in the form of physical contact in painful social situations can put the most vulnerable at greater risk for mental health, anxiety, stress, depression, and even suicidal behavior. Suicide represents a possible scenario of very extreme situations, but the lack of social support can worsen the conditions, even those of family members and survivors (Entilli et al., 2021).

In line with this, Entilli et al. (2021) highlights the importance of social support and suggest the importance of further delving into what specific areas are to be investigated while evaluating social support in bereaved individuals.

For example, Cipolletta et al. (2022) indicate the importance of studying new possible ways of social support also through the use of new technologies. The authors (Cipolletta et al., 2022) indicate how live-chats can represent, for example, in the case of survivors, a valid form of social support to be accessed as a first resource in order to obtain useful information to create the meaning of one’s experience.

The application of new technologies can be a valuable help in reaching more people and offering more help to provide social support. The main challenge of the future will be to be able to integrate new technologies, supported by neuroscientific evidence in order to create new ways of social support. The need to seek new types of effective social support, i.e., not just physical, for the most vulnerable, such as youth and the elderly, is critical in the coming months when social habits may be more restricted (Jawaid, 2020). For example, through an interdisciplinary approach, it could be possible to enhance computer and technological knowledge to implement new realities not based on physical mechanisms (for example, the use of apps, wearable devices) but which help to feel supported thanks to digital innovation.

Research in the field of neuroscience and psychology can contribute to this international debate of the scientific community in an interdisciplinary approach to understand how to reduce the discomfort of physical distancing in social relationships.

## Author Contributions

Both authors conceived the contents, wrote, and revised the manuscript.

## Conflict of Interest

The authors declare that the research was conducted in the absence of any commercial or financial relationships that could be construed as a potential conflict of interest.

## Publisher’s Note

All claims expressed in this article are solely those of the authors and do not necessarily represent those of their affiliated organizations, or those of the publisher, the editors and the reviewers. Any product that may be evaluated in this article, or claim that may be made by its manufacturer, is not guaranteed or endorsed by the publisher.

## References

[B1] Armitage R. Nellums L. B. (2020). COVID-19 and the consequences of isolating the elderly. *Lancet Public Health* 5:e256. 10.1016/S2468-2667(20)30061-X PMC7104160 32199471

[B2] Bozzatello P. Morese R. Valentini M. C. Rocca P. Bosco F. Bellino S. (2019). Autobiographical memories, identity disturbance and brain functioning in patients with borderline personality disorder: an fMRI study. *Heliyon* 5:e01323. 10.1016/j.heliyon.2019.e01323 30949597 PMC6430005

[B3] Cacioppo J. T. Cacioppo S. Boomsma D. I. (2014). Evolutionary mechanisms for loneliness. *Cogn. Emot.* 28 3–21. 10.1080/02699931.2013.837379 24067110 PMC3855545

[B4] Cipolletta S. Entilli L. Bettio F. De Leo D. (2022). Live-chat support for people bereaved by suicide. *Crisis* 43 98–104. 10.1027/0227-5910/a000759 33565355

[B5] Cipolletta S. Gris F. (2021). Older people’s lived perspectives of social isolation during the first wave of the COVID-19 pandemic in Italy. *Int. J. Environ. Res. Public Health* 18:11832. 10.3390/ijerph182211832 34831586 PMC8618043

[B6] Courtney A. L. Meyer M. L. (2020). Self-other representation in the social brain reflects social connection. *J. Neurosci.* 40 5616–5627. 10.1523/JNEUROSCI.2826-19.2020 32541067 PMC7363469

[B7] Entilli L. Leo D. Aiolli F. Polato M. Gaggi O. Cipolletta S. (2021). Social Support and help-seeking among suicide bereaved: a study with Italian Survivors. *Omega.* [Epub ahead of print]. 10.1177/00302228211024112 34128417

[B8] Fabris M. A. Marengo D. Longobardi C. Settanni M. (2020). Investigating the links between fear of missing out, social media addiction, and emotional symptoms in adolescence: the role of stress associated with neglect and negative reactions on social media. *Addict. Behav.* 364–372. 10.1016/j.addbeh.2020.106364 32145495

[B9] Fehr E. Gächter S. (2000). Fairness and retaliation: the economics of reciprocity. *J. Econ. Perspect.* 14 159–181.

[B10] Galić M. Mustapić L. Šimunić A. Sić L. Cipolletta S. (2020). COVID-19 related knowledge and mental health: case of Croatia. *Front. Psychol.* 11:567368. 10.3389/fpsyg.2020.567368 33324280 PMC7726852

[B11] Gerfo E. L. Gallucci A. Morese R. Vergallito A. Ottone S. Ponzano F. (2019). The role of ventromedial prefrontal cortex and temporo-parietal junction in third-party punishment behavior. *Neuroimage* 200 501–510. 10.1016/j.neuroimage.2019.06.047 31233906

[B12] Gleason G. E. Iida M. Shrout P. E. Bolger N. (2008). Receiving support as a mixed blessing: evidence for dual effects of support on psychological outcomes. *J. Pers. Soc. Psychol.* 94 824–838. 10.1037/0022-3514.94.5.824 18444741 PMC3898885

[B13] Groarke J. M. Berry E. Graham-Wisener L. McKenna-Plumley P. E. McGlinchey E. Armour C. (2020). Loneliness in the UK during the COVID-19 pandemic: cross-sectional results from the COVID-19 Psychological Wellbeing Study. *PLoS One* 15:e0239698. 10.1371/journal.pone.0239698 32970764 PMC7513993

[B14] Gryksa K. Neumann I. D. (2022). Consequences of pandemic-associated social restrictions: role of social support and the oxytocin system. *Psychoneuroendocrinology* 135:105601. 10.1016/j.psyneuen.2021.105601 34837776 PMC8605825

[B15] Guéguen N. Fischer-Lokou J. (2003). Tactile contact and spontaneous help: an evaluation in a natural setting. *J. Soc. Psychol.* 143 785–787. 10.1080/00224540309600431 14658752

[B16] Heatley Tejada A. Dunbar R. I. M. Montero M. (2020). Physical contact and loneliness: being touched reduces perceptions of loneliness. *Adapt. Hum. Behav. Physiol.* 6, 292–306. 10.1007/s40750-020-00138-0 32837856 PMC7250541

[B17] Holt-Lunstad J. Smith T. B. Baker M. Harris T. Stephenson D. (2015). Loneliness and social isolation as risk factors for mortality: a meta-analytic review. *Perspect. Psychol. Sci.* 10 227–237. 10.1177/1745691614568352 25910392

[B18] Jakubiak B. K. Feeney B. C. (2017). Affectionate touch to promote relational, psychological, and physical well-being in adulthood: a theoretical model and review of the research. *Pers. Soc. Psychol. Rev.* 21 228–252. 10.1177/1088868316650307 27225036

[B19] Jarvis C. I. Van Zandvoort K. Gimma A. Prem K. CMMID COVID-19 Working Group Klepac P. (2020). Quantifying the impact of physical distance measures on the transmission of COVID-19 in the UK. *BMC Med.* 18:124. 10.1186/s12916-020-01597-8 32375776 PMC7202922

[B20] Jawaid A. (2020). Protecting older adults during social distancing. *Science* 368:145. 10.1126/science.abb7885 32273460

[B21] Kraus M. W. Huang C. Keltner D. (2010). Tactile communication, cooperation, and performance: an ethological study of the NBA. *Emotion* 10 745–749. 10.1037/a0019382 21038960

[B22] Kumar A. Salinas J. (2021). The long-term public health impact of social distancing on brain health: topical review. *Int. J. Environ. Res. Public Health* 18:7307. 10.3390/ijerph18147307 34299756 PMC8305633

[B23] Lam J. A. Murray E. R. Yu K. E. Ramsey M. Nguyen T. T. Mishra J. (2021). Neurobiology of loneliness: a systematic review. *Neuropsychopharmacology* 46 1873–1887. 10.1038/s41386-021-01058-7 34230607 PMC8258736

[B24] Lin S. Liu D. Niu G. Longobardi C. (2020). Active social network sites use and loneliness: the mediating role of social support and self-esteem. *Curr. Psychol.* [Epub ahead of print]. 10.1007/s12144-020-00658-8

[B25] Lindsey E. W. (2020). Relationship context and emotion regulation across the life span. *Emotion* 20 59–62. 10.1037/emo0000666 31961179

[B26] Longobardi C. Morese R. Fabris M. A. (2020). COVID-19 emergency: social distancing and social exclusion as risks for suicide ideation and attempts in adolescents. *Front. Psychol.* 3115:551113. 10.3389/fpsyg.2020.551113 33329182 PMC7710515

[B27] Marengo D. Settanni M. Fabris M. A. Longobardi C. (2021). Alone, together: fear of missing out mediates the link between peer exclusion in WhatsApp classmate groups and psychological adjustment in early-adolescent teens. *J. Soc. Pers. Relat.* 38 1371–1379. 10.1177/0265407521991917

[B28] McClelland H. Evans J. J. Nowland R. Ferguson E. O’Connor R. C. (2020). Loneliness as a predictor of suicidal ideation and behaviour: a systematic review and meta-analysis of prospective studies. *J. Affect. Disord.* 274 880–896. 10.1016/j.jad.2020.05.004 32664029

[B29] Montoya P. Larbig W. Braun C. Preissl H. Birbaumer N. (2004). Influence of social support and emotional context on pain processing and magnetic brain responses in fibromyalgia. *Arthritis Rheum.* 50 4035–4044. 10.1002/art.20660 15593181

[B30] Morese R. Stanziano M. Palermo S. (2018). Commentary: metacognition and perspective-taking in Alzheimer’s disease: a mini-review. *Front. Psychol.* 9:2010. 10.3389/fpsyg.2018.02010 30405494 PMC6204392

[B31] Morese R. Lamm C. Bosco F. M. Valentini M. C. Silani G. (2019a). Social support modulates the neural correlates underlying social exclusion. *Soc. Cogn. Affect. Neurosci.* 14 633–643. 10.1093/scan/nsz033 31056647 PMC6688450

[B32] Morese R. Palermo S. Defedele M. Nervo J. Borraccino A. (2019b). “Vulnerability and social exclusion: risk in adolescence and old age,” in *The New Forms of Social Exclusion*, eds Morese R. Palermo S. (London: IntechOpen). 10.5772/intechopen.85463

[B33] Morese R. Longobardi C. (2020). Suicidal ideation in adolescence: a perspective view on the role of the ventromedial prefrontal cortex. *Front. Psychol.* 11:713. 10.3389/fpsyg.2020.00713 32351433 PMC7174734

[B34] Morese R. Palermo S. (2020). Altruistic punishment and impulsivity in Parkinson’s Disease: a social neuroscience perspective. *Front. Behav. Neurosci.* 14:102. 10.3389/fnbeh.2020.00102 32792921 PMC7385270

[B35] Morese R. Palermo S. Raffaella F. (2020). *Social Isolation - An Interdisciplinary View.* London: IntechOpen.

[B36] Morese R. Rabellino D. Sambataro F. Perussia F. Valentini M. C. Bara B. G. (2016). Group membership modulates the neural circuitry underlying third party punishment. *PLoS One* 11:e0166357. 10.1371/journal.pone.0166357 27835675 PMC5106004

[B37] Ozbay F. Johnson D. C. Dimoulas E. Morgan C. A. Charney D. Southwick S. (2007). Social support and resilience to stress: from neurobiology to clinical practice. *Psychiatry* 4 35–40. 20806028 PMC2921311

[B38] Palermo S. Carassa A. Morese R. (2020). Editorial: perspective-taking, self-awareness and social cognition in neurodegenerative disorders, cerebral abnormalities and Acquired Brain Injuries (ABI): a neurocognitive approach. *Front. Psychol.* 11:614609. 10.3389/fpsyg.2020.614609 33329288 PMC7733928

[B39] Palermo S. Stanziano M. Morese R. (2018). Commentary: anterior cingulate cortex and response conflict: effects of frequency, inhibition and errors. *Front. Behav. Neurosci.* 12:171. 10.3389/fnbeh.2018.00171 30138490 PMC6092509

[B40] Parisi R. Lagomarsino F. Rania N. Coppola I. (2021). Women face to fear and safety devices during the COVID-19 Pandemic in Italy: impact of physical distancing on individual responsibility, intimate, and social relationship. *Front. Public Health* 9:622155. 10.3389/fpubh.2021.622155 33777882 PMC7994331

[B41] Perlman D. Peplau L. A. (1984). “Loneliness research: a survey of empirical findings,” in *Preventing the Harmful Consequences of Severe and Persistent Loneliness*, eds Peplau L. A. Goldston S. E. (Rockville, MD: National Institute of Mental Health), 13–46.

[B42] Rabellino D. Morese R. Ciaramidaro A. Bara B. Bosco F. M. (2016). Third-Party punishment: altruistic and anti-social behaviors in in-group and out-group settings. *J. Cogn. Psychol*. 28 1–10. 10.1080/20445911.2016.1138961

[B43] Rania N. Coppola I. (2021). Psychological impact of the lockdown in italy due to the COVID-19 outbreak: Are there gender differences? *Front. Psychol.* 12:567470. 10.3389/fpsyg.2021.567470 33796039 PMC8007861

[B44] Rico-Uribe L. A. Caballero F. F. Martín-María N. Cabello M. Ayuso-Mateos J. L. Miret M. (2018). Association of loneliness with all-cause mortality: a meta-analysis. *PLoS One* 13:e0190033. 10.1371/journal.pone.0190033 29300743 PMC5754055

[B45] Suvilehto J. T. Nummenmaa L. Harada T. Dunbar R. I. Hari R. Turner R. (2019). Cross-cultural similarity in relationship-specific social touching. *Proc. R. Soc. B* 286:20190467. 10.1098/rspb.2019.0467 31014213 PMC6501924

[B46] Thomas P. A. Kim S. (2021). Lost Touch? Implications of Physical Touch for Physical Health. *J. Gerontol. Ser. B Psychol. Sci. Soc. Sci.* 76 e111–e115. 10.1093/geronb/gbaa134 32845008 PMC7499739

[B47] Thorp H. H. (2020). Time to pull together. *Science* 367:1282. 10.1126/science.abb7518 32179702

[B48] Tomasello M. (2009). *Why We Cooperate.* (Cambridge, MA: The MIT Press), 208.

[B49] Winterton A. Rødevand L. Westlye L. T. Steen N. E. Andreassen O. A. Quintana D. S. (2020). Associations of loneliness and social isolation with cardiovascular and metabolic health: a systematic review and meta-analysis protocol. *Syst. Rev.* 9:102. 10.1186/s13643-020-01369-8 32366295 PMC7199368

[B50] World Health Organization [WHO] (2020). Available online at: https://www.who.int/westernpacific/emergencies/covid-19/information/physical-distancing

